# Modifiable Cardiovascular Disease Risk Factors among Indigenous Populations

**DOI:** 10.1155/2014/547018

**Published:** 2014-02-06

**Authors:** Adam A. Lucero, Danielle M. Lambrick, James A. Faulkner, Simon Fryer, Michael A. Tarrant, Melanie Poudevigne, Michelle A. Williams, Lee Stoner

**Affiliations:** ^1^School of Sport and Exercise, Private Bag 756, Massey University, Wellington 6140, New Zealand; ^2^Institute of Food Nutrition and Human Health, Massey University, Wellington 6140, New Zealand; ^3^Faculty of Applied Sciences, University of Gloucestershire, The Park, Cheltenham, Gloucestershire GL50 2RH, UK; ^4^Warnell School of Forestry, University of Georgia, Athens, GA 30602, USA; ^5^Health & Fitness Management Program, Office of the Dean, Clayton State University, Morrow, GA 30260, USA; ^6^Department of Epidemiology, Harvard School of Public Health, Boston, MA 02115, USA

## Abstract

*Objective*. To identify modifiable cardio-metabolic and lifestyle risk factors among indigenous populations from Australia (Aboriginal Australians/Torres Strait Islanders), New Zealand (Māori), and the United States (American Indians and Alaska Natives) that contribute to cardiovascular disease (CVD). *Methods*. National health surveys were identified where available. Electronic databases identified sources for filling missing data. The most relevant data were identified, organized, and synthesized. *Results*. Compared to their non-indigenous counterparts, indigenous populations exhibit lower life expectancies and a greater prevalence of CVD. All indigenous populations have higher rates of obesity and diabetes, hypertension is greater for Māori and Aboriginal Australians, and high cholesterol is greater only among American Indians/Alaska Natives. In turn, all indigenous groups exhibit higher rates of smoking and dangerous alcohol behaviour as well as consuming less fruits and vegetables. Aboriginal Australians and American Indians/Alaska Natives also exhibit greater rates of sedentary behaviour. *Conclusion*. Indigenous groups from Australia, New Zealand, and the United States have a lower life expectancy then their respective non-indigenous counterparts. A higher prevalence of CVD is a major driving force behind this discrepancy. A cluster of modifiable cardio-metabolic risk factors precede CVD, which, in turn, is linked to modifiable lifestyle risk factors.

## 1. Introduction

**Figure 1:**
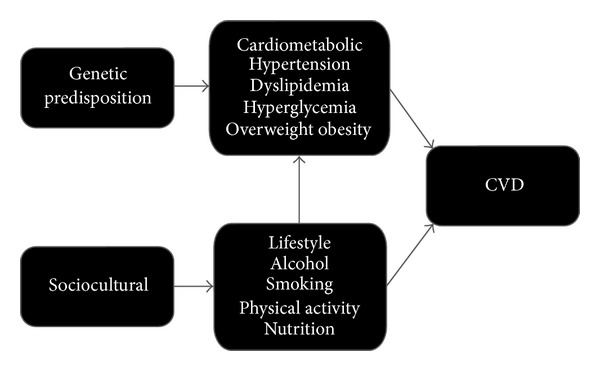
Causation pathway for cardiovascular disease (CVD) [4].

Cardiovascular disease (CVD) is considered the primary influencing factor in the life expectancy discrepancy between indigenous and nonindigenous groups in many countries [1]. Preceding CVD, many groups exhibit a cluster of cardiometabolic risk factors, which, in turn, is linked with a number of modifiable lifestyle risk factors (Figure 1). Multiple studies have revealed that modifiable risk factors are responsible for a large number of premature deaths due to CVD [2, 3]. Recently, it was reported that the single largest risk factor for cardiovascular mortality in the US was high blood pressure, directly responsible for 45% of all CVD deaths, closely followed by obesity, physical inactivity, high cholesterol, and smoking [2]. Fortunately, many of these metabolic and lifestyle risk factors are modifiable and relatively simple to monitor.

The current review will focus on known modifiable *cardio-metabolic* (overweight obesity, diabetes, high cholesterol, and high blood pressure; see Table 1) and common *lifestyle* (physical inactivity, poor nutrition, dangerous alcohol behaviour, and cigarette smoking; see Table 2) risk factors among indigenous populations from Australia, New Zealand, and the United States. Comparisons will be made with nonindigenous groups, and discussion will focus on the association of lifestyle factors and cardiovascular-metabolic conditions. Recommendations will be provided for measuring and tracking each of these risk factors.

## 2. Methods

### 2.1. Data Sources

Electronic databases included PubMed, Medline, and Google Scholar. All titles were exported to Endnote and checked for duplicates.

### 2.2. Study Inclusion and Exclusion Criteria

National health surveys were identified where available. Electronic databases identified sources for filling missing data. Criteria for inclusion of articles included (a) published in a peer-reviewed English-language journal or government report; (b) contained data from nonindigenous cohorts for use as a comparison group; (c) cited in health science, nursing, medical, or exercise science literature. The largest sample studies containing data for CVD prevalence and mortality published between 2002 and July 2012 were selected to compare data of CVD and lifestyle risk factors and conditions (Tables 1 and 2).

### 2.3. Data Extraction and Data Synthesis

Search terms included Aboriginal Australians, Māori, American Indians/Alaska Natives, indigenous, cardiovascular disease, heart disease, overweight, obesity, diabetes, cholesterol, blood pressure, hypertension, alcohol, physical activity, exercise, nutrition, cigarette smoking, and tobacco.

## 3. Demographics

### 3.1. Australia

For the purpose of this paper, the term indigenous Australian refers to those of Aboriginal origin and Torres Strait Islanders. The indigenous population is estimated to be 2.5% of the total Australian population, approximately 90% of which self identify as Aboriginal, 6% as Torres Strait Islander, and 4% both mixed [5]. The indigenous population is relatively young, with a median age of 20.5 years compared to 36.6 years for the nonindigenous population [5]. Around 26% of indigenous people live in remote areas, compared with only 2% nonindigenous peoples [5], and many continue to maintain a strong connection to their traditional culture, language, and lands.

### 3.2. New Zealand

For the purpose of this paper, the term indigenous New Zealander encompasses those of Māori descent. Māori people comprise 15% of the total population [6], with the predominance (84.4%) residing in urban areas [6]. The Māori population has a median age of 22.7 years compared to 35.9 years for the nonindigenous population [6]. Māori culture continues to be an important thread of New Zealand society with 23.7% of Māori being able to hold a conversation in *te Reo Māori* [6].

### 3.3. United States

For the purpose of this paper, the term indigenous for a person from the United States encompasses American Indians and Alaska Natives. There are approximately 5.2 million reported indigenous Americans in the United States, representing 1.7% of the population, including those of more than one race [7]. The indigenous population is younger than the nonindigenous population (30.3 years compared to (cf.) 36.6 years, resp., [8]), and is varied with 566 federally recognized tribes [9]. These tribal groups often have different histories, unique languages, and varied cultural traditions, reside in numerous geographic regions, and show various degrees of societal assimilation [10]. In 2010, the majority (78%) of indigenous Americans lived outside of native reservation areas [11].

### 3.4. Cardiovascular Disease

CVD covers all diseases and conditions of the heart and blood vessels. Coronary heart disease (CHD), stroke, heart failure, and peripheral vascular disease contribute approximately 30% to the CVD burden in developed countries [12]. In 2001, CVD was the primary cause of death worldwide, with indigenous peoples leading the way [1].

#### 3.4.1. Australia

CVD is the principal cause of death among all ethnic groups in Australia [13]. The 2004-05 National Aboriginal and Torres Strait Islander Health Survey (NATSIHS, the largest health survey of Indigenous Australians, [13]) found age-adjusted rates for CVD that were 30% higher among the indigenous population (22%) compared to the nonindigenous (17%) population, with CVD mortality three times higher (27% cf. 9%, resp.). Indigenous Australians show a marked increase in the prevalence of CVD from around 35 years of age onwards, some 10 years earlier than in the nonindigenous population.

#### 3.4.2. New Zealand

Across all ethnic groups, CVD mortality peaked between 1966 and 70, and since then, death rates have fallen by over 60% in all age and sex groups [14, 15]. However, the decline has been slower among indigenous New Zealanders. Between 1981 and 2004, CVD mortality rates decreased by 43% among indigenous New Zealanders compared to 65% among nonindigenous New Zealanders [14, 15]. Indigenous New Zealanders continue to have a higher prevalence of CVD compared to their nonindigenous counterparts (7% cf. 4%, resp., [16]). Chan et al. [16] found that CVD prevalence begins to rise after age 35 years among all age groups, but the rise in prevalence rate is greater among indigenous New Zealanders.

#### 3.4.3. United States

Among all ethnicities, CVD accounted for 34% of all deaths in 2010 [17], 33% of which occurred before the age of 75 years—well before the average life expectancy of 78 years. According to the National Center for Health Statistics (NCHS) [18], if all forms of major CVD were eliminated, life expectancy would rise by almost 7 years. Despite a lower life expectancy and a slightly higher prevalence of CVD among indigenous versus nonindigenous Americans (23% cf. 21%), CVD mortality is lower (25% cf. 34%) [17]. These discrepant findings may be partially explained by higher rates of mortality among indigenous Americans for tuberculosis (600% higher), alcoholism (510% higher), motor vehicle crashes (229% higher), diabetes (189% higher), unintentional injuries (152% higher), homicide (61% higher), and suicide (62% higher) [19].

## 4. Modifiable Cardiometabolic Metabolic Risk Factors

### 4.1. Obesity

Excess body fat increases the risk of developing a range of health problems, including high blood pressure, diabetes mellitus, and CVD [20–22]. According to results from the Framingham Heart Study [20], age-adjusted relative risk for CVD is increased for overweight and obese men (21% and 46%, resp.) and women (20% and 64%, resp.) when compared with normal weight individuals. Population studies, including those retrieved for the current paper, typically estimate the proportion of people that are obese by calculating an individual's body mass index (BMI). BMI is based on the assumption that the ratio between body mass and height provides an indication of body fatness; however, this often discriminates against individuals (and/or populations) that have a higher proportion of muscle mass. Alternatively, waist circumference, waist-to-height ratio, and waist-to-hip ratio (WHR) take into consideration body-fat distribution, especially central (abdominal) obesity [23]. A recent study compared the predictive power of BMI, waist circumference, waist-to-height ratio, and WHR for diabetes mellitus, hypertension, and dyslipidemia in Australian Aboriginal and Torres Strait Islander adults [24]. WHR was found to have the greatest predictive power. A WHR of ≥0.90 and ≥0.80, for males and, females respectively, is considered optimal. However, studies over the past two decades indicate that the rate of risk for a given WHR differs between ethnic groups; therefore these reference values should not be used to ascertain absolute risk [25].

#### 4.1.1. Australia

After adjusting for age differences between the two populations, the 2004/05 NATSIHS [13] reported that indigenous Australians are 1.2 times more likely to be overweight/obese than nonindigenous Australians (62% cf. 51%). In each age group, the disparity between indigenous and nonindigenous groups was greater for females than for males and was more pronounced within remote geographical areas.

#### 4.1.2. New Zealand

The 2006/07 New Zealand National Health Survey (NZNHS) [26] reported that 36% of adults were overweight and a further 27% were obese. The obesity burden is particularly prevalent among indigenous New Zealanders, with 42% of this population being obese compared to 24% of their white counterparts [26]. However, there was no significant increase reported for either ethnic group between 2002/3 and 2006/07.

#### 4.1.3. United States

An estimated 144,100,000 people or 66% of the total US adult population is overweight or obese [17]. The rates of overweight/obesity are comparable between indigenous and white Americans. However, while indigenous Americans are less likely to be overweight (28%) than white Americans (33%) they are more likely to be obese (42% cf. 31%, resp.). These findings have been corroborated by other studies [10].

### 4.2. Diabetes

Diabetes mellitus is a group of metabolic diseases in which hyperglycaemia results from defective insulin secretion, insulin action, or both [27, 28]. There are several forms of diabetes mellitus, each with a different cause and clinical history. The two most prominent forms are type 1 and type 2 diabetes, which differ according to their underlying pathophysiology, with type 1 often attributed to an autoimmune response and type 2 often related to several lifestyle factor. type 2 diabetes accounts for 90–95% of diabetes cases and is a major risk factor for CVD [17, 29–34]. A meta-analysis [34], encompassing 6,573 subjects found that type 2 diabetes resulted in greater CVD mortality risk (RR 3.42, 95% CI: 2.23 to 5.23) than hypertension (RR 1.57, 95% CI: 1.10 to 2.24) or hypercholesteremia (RR 1.49, 95% CI: 1.05 to 2.10). In turn, diabetes is modified by lifestyle factors, including physical inactivity and poor nutrition, in addition to any genetic predisposition and the natural ageing process [35–39]. Diabetes mellitus risk can be monitored by measuring glucose tolerance or fasting blood glucose, where a fasting blood glucose of <100 mg/dL is considered optimal [40].

#### 4.2.1. Australia

The 2007/08 Australian National Health Survey [41] reported that an estimated 4% of the total population had diagnosed diabetes. According to the 2004/05 NATSIHS [13], the age-standardized prevalence of diabetes/elevated blood glucose (i.e., prediabetes) among indigenous Australians was 3.4 times the rate of that observed in nonindigenous people (12% cf. 4%). The prevalence of diabetes among indigenous Australians increases rapidly after 35 years of age, rising from 10% at age 35–44 years to 32% at age 55 years and over. By contrast, the prevalence increases from 2% to 12% for nonindigenous people. Prevalence rates among indigenous Australians are similar between genders, but those in remote areas are almost twice as likely to have diabetes [13].

#### 4.2.2. New Zealand

The 2006/07 NZNHS [26] reported 5% of the total population was diagnosed with diabetes. Indigenous New Zealanders were twice as likely to be diagnosed with diabetes (8% cf. 4%, resp.). There has been no significant change in diabetes prevalence between 1996/97 and 2006/07 for both indigenous and nonindigenous populations or for males and females [26].

#### 4.2.3. United States

In 2006, an estimated 17,200,000 Americans had diagnosed diabetes, representing 8% of the adult population [17]. A further estimated 6,100,000 had undiagnosed diabetes and 29% had prediabetes with abnormal fasting glucose levels. Diabetes was once rare among indigenous Americans, but the prevalence is rising dramatically with rates almost twice as high when compared with their non-Hispanic white counterparts (15% cf. 8%, resp.) [17].

### 4.3. Cholesterol

The two most common blood lipids are cholesterol and triglycerides. These two blood fats are carried on particles called lipoproteins, the most important of which are low density lipoprotein (LDL) and high density lipoprotein (HDL). Both carry cholesterol, but high levels of LDL cholesterol have been shown to be atherogenic [42–44]. Similarly, low levels of HDL cholesterol are associated with increased CHD morbidity and mortality [45–47]. High HDL cholesterol levels conversely convey reduced risk [45–48]. In population studies, serum total cholesterol is often used as a surrogate for LDL cholesterol levels; however, LDL concentrations confers more predictive value. The best way to determine the true prevalence of high cholesterol in the community is through blood samples [26]. An LDL cholesterol level <100 mg/dL is considered optimal [49].

#### 4.3.1. Australia

The 2004/05 NATSIHS [13] found the prevalence of high age-adjusted serum total cholesterol levels to be similar for indigenous (6%) and nonindigenous (7%) groups. The same report found the prevalence of high total cholesterol levels to drastically increase with age, starting at 4% for both indigenous and nonindigenous groups when aged 35–44 years, rising to 18% and 20%, respectively, aged 55 years or over.

#### 4.3.2. New Zealand

The 2006/07 NZNHS [26] reported that 8% of the total adult (≥15 years) population were currently taking medication for high cholesterol. Men (8%) were significantly more likely than women (6%) to be taking medication for high cholesterol, when standardized for age. Age-standardized cholesterol levels are similar between indigenous and nonindigenous groups. However, the lack of differences in cholesterol between populations may be misleading as the NZNHS data only details the number of individuals on medication for high cholesterol, therefore excluding those who remain undiagnosed.

#### 4.3.3. United States

In 2006, an estimated 102,200,000 people or 47% the total US adult population (≥20) had total cholesterol levels above ≥200 mg/dL, with an estimated 16% registering a cholesterol level ≥240 mg/dL [17]. The prevalence of high total cholesterol (≥240 mg/dL) is substantially greater for indigenous Americans compared to nonindigenous Americans (31% cf. 17%) [17].

### 4.4. Hypertension

Hypertension is a major risk factor for CVD. For every 20 mmHg systolic or 10 mmHg diastolic increase in resting blood pressure there is a twofold increase in risk of death from ischemic heart disease or stroke [50]. Hypertension is associated with shorter overall life expectancy and earlier onset of CVD [51]. According to the WHO, hypertension is likely the leading risk factor for death worldwide [52]. In part, this is because hypertension is common and because management of hypertension is suboptimal [53]. An ideal blood pressure is one with a systolic pressure <120 mmHg and a diastolic pressure <80 mmHg [40].

#### 4.4.1. Australia

In 2004/05, hypertension was the most commonly reported CVD condition among indigenous Australians, with prevalence rates 50% greater than for nonindigenous Australians when adjusted for age (10% cf. 15%, resp.) [13]. Hypertension is of particular concern to remote indigenous groups with an overall prevalence of 6% among urban dwellers versus 10% for remote dwellers. For both ethnic groups prevalence increases dramatically with age, from 12 to 43% for indigenous and 4–33% for nonindigenous groups between the ages 35–44 years and 55+ years, respectively.

#### 4.4.2. New Zealand

In 2006/07 one in seven adults (14%) reported that they were currently taking medication for high blood pressure [26]. After adjusting for age, indigenous New Zealanders were 26% more likely to have elevated blood pressure than the general population.

#### 4.4.3. United States

Data from the 2006 National Health and Nutrition Examination Survey (NHANES) [54] indicate that 34% of US adults over 20 years have hypertension. Rates of hypertension are slightly lower among indigenous (30%) than nonindigenous Americans (33%) [54].

## 5. Modifiable Lifestyle Risk Factors

### 5.1. Nutrition

Poor dietary habits affect multiple cardiovascular risk factors including blood pressure, cholesterol levels, glucose levels, and obesity [55–61]. A diet high in nutrient-rich (vitamins, minerals, antioxidants and fibre) fruits and vegetables can reduce the risk for many leading causes of death [17, 55–57, 62]. In meta-analyses of prospective cohort studies, each daily serving of fruits or vegetables was associated with a 4% lower risk of CHD (RR 0.96, 95% CI: 0.93 to 0.99) and a 5% lower risk of stroke (RR: 0.95, 95% CI 0.92 to 0.97) [56, 57]. Five or more daily servings of fruits and vegetables are recommended for optimal nutrition [17, 62]. Direct observation is considered the “gold standard” for monitoring dietary intake [63–65]. However, this approach can be time consuming and impractical for use in large-scale studies. Alternatively, a food frequency questionnaire (FFQ), including the freely available National Cancer Institute Diet History Questionnaire (http://riskfactor.cancer.gov/dhq2/) [66, 67], allows for assessment of the usual patterns of food intake over an extended period of time [68, 69] and is considerably less burdensome in both time and cost than other measurement tools [70, 71].

#### 5.1.1. Australia

In 2004/05, 78% of the total Australian population consumed two or more servings of vegetables per day compared with just 43% of the indigenous population [13]. Similarly, 53% of the total Australian population consumed two or more servings of fruit compared with just 26% of the indigenous population. Rates of consumption were also reportedly much lower for indigenous groups living in remote locations, wherein 20% reported no daily fruit intake and 15% no daily vegetable intake.

#### 5.1.2. New Zealand

In 2006/07, two out of every three adults (64%) consumed the recommended three or more servings of vegetables each day, and 60% consumed the recommended two or more servings of fruits each day [26]. Consumption of the recommended servings of vegetables and fruits was higher for the nonindigenous (67% and 63%, resp.) than the indigenous population (62% and 56%, resp.).

#### 5.1.3. United States

Daily consumption of fruits and vegetables is poor across the general US population, with one recent study [10] estimating that only 23% of the population consume five or more servings of fruits and vegetables per day, with an even lower rate of consumption among the indigenous population (18%). These data are consistent with other nutritional studies in indigenous communities [72–76].

### 5.2. Alcohol

Accumulating scientific evidence indicates that light to moderate alcohol consumption may significantly reduce the risk of CVD and all-cause mortality [77–79]. However, excessive alcohol intake is toxic to both the heart and overall health [77–79]. In particular, binge drinking, even among otherwise light drinkers, increases cardiovascular events and mortality [77–79]. Alcohol should not be universally prescribed for health enhancement owing to the lack of randomised outcome data and the potential for developing irresponsible drinking habits. The American Heart Association warns those who have never consumed alcohol against initiating such behaviours due to the inability to predict the potential for alcohol abuse [80]. Alcohol intake can be monitored using a food frequency survey (FFQ) (see above).

#### 5.2.1. Australia

The 2004/05 NATSIHS [13] reported a lower prevalence of alcohol consumption among indigenous (49%) than nonindigenous (83%) Australians. However, after standardizing for age, indigenous Australians are just as likely as nonindigenous Australians (15% cf. 14%, resp.) to consume higher than recommended daily intakes of alcohol (≥5 standard drinks/day for males and ≥4 standard drinks/day for females). Increased alcohol consumption was reported to be greatest among remote indigenous Australians (19%). For both groups, the prevalence of high alcohol consumption had risen by 3% since the previous (2001) NATSIHS.

#### 5.2.2. New Zealand

In 2007/08, 85% of the New Zealand population reported that they had consumed alcohol in the past year, with a slightly higher rate among the nonindigenous (90%) than indigenous (85%) population [81]. However, the indigenous population were twice as likely (24% cf. 12%, resp.) to exhibit excessive alcohol consumption behaviours (males: ≥6 standard drinks on one occasion; females: ≥4 standard drinks on one occasion).

#### 5.2.3. United States

In 2006/07, a greater portion of the non-Hispanic white population had consumed alcohol in the past month compared to the indigenous population (55% cf. 60%, resp.) [82]. However, a greater proportion of the indigenous population consumed higher than the recommended levels of alcohol consumption (≥5 standard drinks/day for ≥5 days in the past 30 days) than the nonindigenous population (12% cf. 8%, resp.). It is important to note, however, that tribal diversity has been reported for alcohol drinking tendency [83, 84]. Beals et al. [84] compared two culturally and geographically distinct tribes and found that current drinking rates were higher for a Northern Plains tribe than for a Southwest tribe. Gender differences have also been demonstrated, with May and Gossage [85] reporting higher levels of binge drinking among Northern tribe males than females (3 days cf. 1.3 days of drinking ≥5 standard drinks in the past 30 days, resp.).

### 5.3. Physical Activity

It has been estimated that physical inactivity is responsible for 12% of the global burden of myocardial infarction [86]. Regular physical activity reduces CVD risk in its own right and also improves CVD risk factors such as obesity, hypertension, dyslipidemia, and type 2 diabetes [87–92]. The American College of Sports Medicine (ACSM) recommends at least 30 minutes of moderate-intensity physical activity (e.g., walking briskly, dancing, swimming, and bicycling) at least 5 days a week [93]. A number of tools have been developed to measure physical activity, ranging from objective measures, such as accelerometry, to subjective questionnaires [94]. While questionnaires are prone to technical error, they are inexpensive and practical for use in population studies and can provide information about physical activity type and context [94]. The International Physical Activity Questionnaire (IPAQ) (http://www.ipaq.ki.se/ipaq.htm) is a freely available, cross-national monitoring tool which has been validated for use in adults [95–99] and children [100–103].

#### 5.3.1. Australia

In 2004-05 an estimated one in three people (33%) in Australia were sedentary, with an even higher rate (51%) among the indigenous population [13]. Physical inactivity contributes an estimated 7% of Australia's disease burden and 10% of all deaths [104] and accounts for 12% of the health gap between indigenous and nonindigenous Australians [105]. Despite the publicized importance of physical activity levels have remained stagnant in recent National Health Surveys [5, 13].

#### 5.3.2. New Zealand

In 2006/07, half of all adults reported that they met physical activity guidelines (≥30 mins/day on most days), with 15% of all adults identified as sedentary [26]. No significant differences were reported between indigenous and nonindigenous populations. For both ethnic groups, men were more likely to be physically active than women (55% cf. 48%, resp.), and for both groups physical activity levels remained constant between 2002/03 and 2006/07 national surveys. However, while similar physical activity levels have been reported for indigenous and nonindigenous groups, large-scale studies using validated instruments are limited. The national surveys collect physical activity data using a New Zealand version of the IPAQ. Only one study has tested the validity of the NZPAQ and reported poorer accuracy when used on Māori and Pasifika compared to European/other [106].

#### 5.3.3. United States

The 2007 US National Health Interview Survey [107] estimated that 39% of all adults were sedentary, with a higher prevalence among women (41%) versus men (37%). Compared to the nonindigenous population, the indigenous population had a higher rate of sedentary behaviour (40% cf. 37%, resp.) and a lower rate of meeting prescribed physical activity (30 min/day on most days) (10% cf. 12%, resp.). These data agree with other studies showing lower rates of physical activity among indigenous Americans compared to the general population [10, 108–113].

### 5.4. Tobacco Use

Cigarette smoking, which is estimated to kill five million people worldwide each year [117], has been established as a risk factor for CVD since the 1940s [118]. The relationship between smoking and CVD is resultant upon the interaction of multiple mechanisms which contribute to atherosclerosis, vascular injury, vascular dysfunction, and thrombosis, although these precise mechanisms are largely unknown [119]. Cigarette smoking increases the incidence of CVD in a dose-dependent manner [120–122], with even occasional smoking increasing the risk of CVD [123]. Conversely, long-term prospective studies have demonstrated considerable mortality risk reduction with smoking cessation [124–126].

#### 5.4.1. Australia

In 2004/05, an estimated one in five people (21%) in Australia were smoking cigarettes daily [13]. Among the indigenous population, smoking rates are higher for those living in remote (52%) versus nonremote (49%) areas and particularly for males living in remote areas (58% for males cf. 47% for females). For both ethnic groups, daily smoking prevalence declined between 2001 and 2004/05, decreasing from 49% to 46% and from 22% to 21% among indigenous and nonindigenous populations, respectively.

#### 5.4.2. New Zealand

In 2006/07, 20% of the New Zealand population were current cigarette smokers, with a prevalence rate that is twice as high among the indigenous (38%, age adjusted) compared to the nonindigenous population [26]. After adjusting for age, indigenous women were more than twice as likely to be smokers than women in the total population, while indigenous men were 1.5 times more likely to smoke than men in the total population. The prevalence of smoking decreased from 23% in 2002/03 to 19% in 2006/07 among the total population and from 47% to 38% among the indigenous population.

#### 5.4.3. United States

Between 2000 and 2004, cigarette smoking resulted in an estimated 443,000 premature deaths in the US each year [127]. In adults aged 35 years or over, 33% of these deaths were related to CVD. From 1965 to 2007, smoking in the US declined by 50% among people aged 18 years or over [128]. However, despite this progress, in 2008 an estimated 21% of the total US population were current cigarette smokers [107]. The prevalence of cigarette smoking was higher among indigenous Americans at 23%, increasing to 31% when mixed race indigenous Americans were included. There also appear to be notable differences between tribes [10, 129, 130].

## 6. Discussion

There are more than 370 million indigenous people in 70 countries worldwide. Indigenous peoples are not monolithic; there is significant variation between and within peoples in terms of worldview, political forces, education, socioeconomic status, living conditions, and familial factors. However, many indigenous groups do share a striking commonality, a discrepancy in life expectancy when compared to their nonindigenous counterparts. Three such examples can be seen in the life expectancy of indigenous groups in Australia, New Zealand, and America. A higher prevalence of CVD may be considered the driving force behind this discrepancy [1], which is being fuelled by lifestyle and subsequent cardiometabolic risk factors.

The indigenous populations from each of these nations exhibit a cluster of cardiometabolic conditions. Compared to their respective nonindigenous counterparts, all three indigenous groups have higher rates of obesity and diabetes, hypertension is greater for the indigenous populations of New Zealand and Australia, and high cholesterol is greater among indigenous groups in the United States. While each of these conditions has independently been shown to accelerate CVD [131–134], the effects are also thought to be additive [34]. Poor lifestyle choices may precede and contribute to these cardiometabolic outcomes. Compared to their nonindigenous counterparts, all three indigenous groups exhibit higher rates of smoking and dangerous alcohol behaviour, as well as lower consumption of fruits and vegetables. The indigenous groups of Australia and the United States also exhibit greater rates of sedentary behaviour, while there remains a need to collect valid physical activity data in New Zealand [106].

Holistic strategies, which recognize the complex interactions between lifestyle factors, may assist in promoting positive changes. For example, a recent systematic review and meta-analysis [135] reported that physical activity interventions have had only a small effect on children's overall activity levels. This implies that lifestyle strategies to promote physical activity should be sensitive to total daily physical activity as well as other lifestyle factors, including nutrition and sleep behaviour, each of which may be influenced by increased physical activity levels and may affect cardiometabolic outcomes [136, 137].

In order to maximize potential positive outcomes, strategies which aim to promote positive changes in lifestyle should not only be physiologically appropriate; they should also be sensitive to sociocultural norms. For example, within Australia an indigenous person's connections to family, ancestors, the wider community, and the land are very important to the choices they make about all aspects of their lives [138]. Exercising alone for personal benefit may prevent a person from spending time with family and loved ones, and this may be seen as superficial. Similarly, the Māori people of New Zealand show a decided preference for physical activities which involve whanaungatanga/kotahitanga (a team environment), a forum to experience feelings of whanau (extended family) [139]. In this regard an argument can be made that appropriate physical activity prescription can be used as a vehicle to experience, discover, and reconnect to indigenous cultural heritage [140]. However, it must also be recognized that sociocultural norms may substantially differ by group, including within a given nation. For example, in the United States there are 566 federally recognized tribes [9], with different histories, unique languages, varied cultural traditions, and various degrees of societal assimilation [10].

Even within a given nation, strategies to promote lifestyle changes must be specific to a group, not to the population as a whole, especially when a geographical area includes different language and culturally distinct groups [141]. Diversity competence involves knowledge, skills, and abilities that enable a researcher to deal with a specific population. *The National Standards for Culturally and Linguistically Appropriate Services in Health and Health Care *(the *National CLAS Standards*) [142] in the United States intend to advance health equity, improve quality, and help eliminate health care disparities by providing a blueprint for individuals and health and health care organizations to implement culturally and linguistically appropriate services. Adoption of these standards is highly recommended for health care providers interested in respecting such diversity competences.

## 7. Limitations

To ensure reliable comparisons between ethnic groups, the largest data sources available were utilized for a given cohort (nation), where available. Wherever large data sets were not available, reliable sets of relevant data were used to consolidate thought and formulate a comprehensive picture. All data used in the current publication spanned from 2002 to 2012. This would have introduced selection and methodological and historical bias, limiting our ability to make accurate comparisons across nations. Furthermore, the preponderance of the literature on the health of indigenous populations is focused on describing or understanding problems [143], rather than on testing the effectiveness of potential solutions. Further studies are required to determine causality between lifestyle and cardiometabolic risk factors and to determine whether causality is moderated by ethnicity.

## 8. Implications

This review has described the relationship between common lifestyle choices, cardiometabolic conditions (i.e., lifestyle-related disease), and CVD. It is evident that disparities in CVD prevalence, mortality, and associated risk factors exist between indigenous and nonindigenous populations. Causality, however, has yet to be conclusively determined and is essential if we are to develop effective solutions for decreasing disease burden in a number of groups. While the described model will assist future research focusing on indigenous health outcomes, it must also be recognised that such research must be sensitive to differences in culture between indigenous groups within a country, in addition to being sensitive to national cultural norms.

## Figures and Tables

**Table 1:** Prevalence of cardio-metabolic risk factors among adults.

Group	Population		Life expect. yrs	CVD		Body weight			High cholest. * * %	HT	References
	million	%		Prev. %	Mortality %	Over %	Obese %	Diabetes %		%	
AU	20.8	100	81	17	9	29	22	4	7	10	
White AU	20.3	98	81	17	9	29	22	4	7	10	[13, 114, 115]
Indigenous AU	0.52	2.5	62	22	27	35	27	12	6	15	
NZ	4.03	100	80	5	31	36	27	5	8	14	
White NZ	2.61	68	81	4	32	32	24	4	8	13	[6, 14, 16, 26, 116]
Indigenous NZ	0.57	15	73	7	32	32	42	8	9	17	
U.S.	309	100	78	21	34	33	33	8	16	34	
White U.S.	309	100	78	21	34	33	31	6	17	33	[7, 17, 19, 54, 107]
Indigenous U.S.	5.22	1.7	75	23	25	28	42	15	31	30	

CVD: cardiovascular disease; HT: Hypertension.

Notes: a body mass index (BMI) ≥ 25.0 kg/m^2^ is considered overweight, ≥30.0 kg/m^2^ is considered obese.

AU: CVD, cholesterol, diabetes (includes high sugar levels), and body weight data are self-reported and age-adjusted for adults ≥ 18 yrs [13].

NZ: diabetes = physician diagnosed; high cholesterol: individuals medicated for high total cholesterol [26]; HT = currently taking prescribed blood pressure medication [26]; HT, cholesterol, diabetes, and body weight data are for adults aged ≥ 15 [26]; CVD data are age-adjusted for adults ≥18 [14, 16].

US: diabetes = physician diagnosed; high cholesterol = ≥240 mg/dL [17]; HT = defined as SBP ≥ 140 mmHg and/or DBP ≥ 90 mmHg, use of antihypertensive medication, or physician diagnosed [17]; CVD, hypertension, diabetes, and body weight data are age-adjusted for adults ≥20 [17].

**Table 2:** Prevalence of modifiable lifestyle risk factors.

Group	Activity behaviour		Nutrition		Alcohol behaviour		Smokers * *%	References
	Sedentary %	Prescribed %	Veg. % ≥2 day	Fruit % ≥2 day	Any %	Risky %		
AU	33	33	78	52	83	14	21	
White AU	33	33	78	52	83	14	21	[13]
Indigenous AU	51	21	43	26	49	15	46	
New Zealand	15	51	64	60	85	13	20	
White NZ	14	51	67	63	90	12	19	[26, 81]
Indigenous NZ	14	51	62	56	85	24	38	
U.S.	39	11	23		55	7	21	
White U.S.	37	12	N/A		60	8	22	[10, 17, 82, 107]
Indigenous U.S.	40	10	17		48	12	24	

AU: sedentary activity behavior = <50 mins/week, moderate: >800 mins/week, for adults ≥15 yrs [13]; risky alcohol behavior = ≥ 5 standard drinks/day for males (or ≥15/week) and ≥4 for females (or ≥8/week) for adults aged ≥ 18 yrs [13]; smoker: any type of tobacco consumption [13]; smoking, activity and nutrition data are age-adjusted for adults ≥18 yrs [13].

NZ: prescribed activity behavior = recommended ≥30 mins/day most days or at least 150 mins/week, sedentary activity behavior = <30 mins/week [26]; risky alcohol behavior = weekly binge (≥6 standard drinks on one occasion for males and ≥4 for females) drinking, age-adjusted for adults aged 16–64 [81]; smoker = cigarette smoking [26]; vegetable = ≥3 servings/day [26]; smoking, activity and nutrition date are age-adjusted for adults aged 16–64 [26].

US: prescribed activity behavior = ≥30 mins/day most days or at least 150 mins/week (self-reported, ≥18 yrs), age-adjusted for adults aged ≥18 yrs [107]; nutrition = ≥5 servings/day of vegetables/fruit, age-adjusted for adults aged ≥18 yrs [10]; risky alcohol behaviour = ≥ 5 standard drinks/day on ≥5 days in past 30 days [82]; smoking = cigarette smoking, age-adjusted for adults aged ≥20 [107].
